# Safety of In‐Hospital Delay of Appendectomy in Elderly Patients—A Retrospective Analysis of 525 Consecutive Patients Aged 65 and Older Undergoing Surgery for Suspected Appendicitis

**DOI:** 10.1002/wjs.70178

**Published:** 2025-12-05

**Authors:** Matthias C. Schrempf, Stefan Schiele, Matthias Anthuber, Lena Anthuber, Michael Hoffmann, Florian Sommer, Andrea Mair

**Affiliations:** ^1^ Department of General, Visceral and Transplantation Surgery University Hospital Augsburg Augsburg Germany; ^2^ Department of Computational Statistics and Data Analysis Institute of Mathematics, University of Augsburg Augsburg Germany

## Abstract

**Background:**

Recent retrospective and prospective studies have demonstrated the safety of delayed surgery for acute appendicitis. However, evidence regarding delayed surgery in elderly patients is limited, and it is unclear whether it is safe to delay appendectomy in this patient population.

**Methods:**

The outcomes of patients aged 65 years and older who underwent appendectomy for suspected appendicitis at a single institution were reviewed and analyzed. The outcomes investigated were perforation rate and complication rate. Multivariable logistic regression analysis was performed to adjust for risk factors and calculate adjusted odds ratios (aOR) for in‐hospital delay.

**Results:**

A total of 525 patients aged 65 years and older underwent appendectomy for suspected appendicitis. The perforation rate was 44.4% (233 of 525) and the complication rate was 25.1%. The multivariable analysis showed no association between a waiting time of more than 12 h compared to less than 12 h and perforation rate (aOR 0.96; 95% CI 0.55–1.70; *p* = 0.90) or complication rate (aOR 0.93; 95% CI 0.49–1.76; *p* = 0.83). A risk factor for perforation in the multivariable analysis was an elevated CRP level ≥ 50 mg/L and risk factors for complications were suspected perforation on preoperative imaging (*p* = 0.004), anticoagulant use (*p* = 0.04), and CRP levels ≥ 150 mg/L (*p* < 0.001).

**Conclusion:**

This large retrospective analysis showed that it is safe to delay surgery by 12 h in patients aged 65 years and older. Delayed surgery was not associated with a higher rate of perforation or complications after adjusting for risk factors. These results open the possibility of optimizing coagulation or possible comorbidities in elderly patients before surgery or postponing surgery when more critical cases require more urgent treatment. In patients with suspected perforation on imaging, the decision to delay surgery should me made with caution, taking into account the patient's overall presentation, comorbidities, and vital signs.

## Graphical abstract

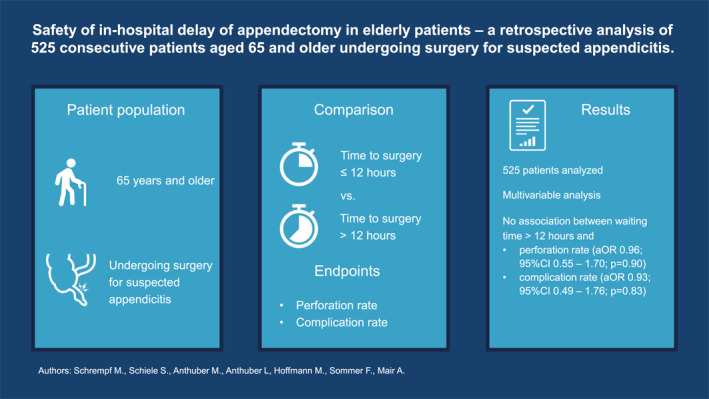

## 1 Introduction

Acute appendicitis is one the most frequent causes of abdominal pain and one of the most common surgical emergencies worldwide [1, 2]. Patients between the ages of 10 and 30 are most commonly affected, but many studies describe a second, but lower incidence peak in older patients [3, 4].

Traditionally, early appendectomy has been recommended for appendicitis due to concerns of progression to perforation but the more than a 100‐year‐old assumption that untreated appendicitis would inevitably leads to perforation has recently been challenged [5, 6, 7, 8, 9, 10, 11].

Large retrospective studies and two small prospective trials have demonstrated that delayed surgery for appendicitis is safe and that an in‐hospital delay of 12–24 h does not result in an increased risk for perforation and complications [8, 9, 12, 13]. Most guidelines agree that an in‐hospital delay of up to 24 h for suspected appendicitis is acceptable, but there is still an ongoing debate whether it is safe to delay surgery in the elderly population or if comorbidities are present. Certain guidelines advocate against the delay of surgical treatment in patients aged 65 and older due to limited data [14]. The *European Association for Endoscopic Surgery* (EAES) rapid guideline regarding appendicitis in the elderly does not comment on the safety of delaying surgery in this patient population [15].

Elderly represents a distinct population in general surgery due to their diminished functional reserve and biological frailty [16]. These factors modulate their resilience to surgical stress, increase their susceptibility to adverse postoperative outcomes, and complicate decision‐making regarding the timing of interventions such as surgery for acute appendicitis. Although several measurement such as hand‐grip strength and sarcopenia have been suggested as predictors of surgical outcomes in the elderly, these are usually impractical to assess in an emergency setting [17, 18].

Older patients typically represent a smaller population in most studies, and several studies show that elderly patients with acute appendicitis differ from younger patients with respect to several characteristics [4, 19, 20]. These include a longer duration of symptoms before seeking medical attention, a higher rate of perforation and complications, and a longer hospital stay [2, 4, 19, 20]. Another finding that complicates diagnosis and treatment is that the rate of unsuspected malignancies of the appendix is higher in older patients than in younger patients, and that older patients often present with atypical symptoms, making diagnosis of the disease based on clinical examination and ultrasound difficult and often requiring additional diagnostic measures [2, 4, 20].

The perforation rate for acute appendicitis in the general population is between 16% and 40%, and although mortality from acute appendicitis is low, perforated appendicitis is still associated with higher mortality rates than uncomplicated appendicitis [2, 5]. Advanced age has been identified as a risk factor for perforation in several studies, but there is no data on whether perforation is more common in this patient group if surgery is delayed [4, 19, 20, 21]. Due to the limited evidence in older patients and the potential association between perforated appendicitis and higher mortality, many guidelines caution against delaying surgery and recommend early appendectomy for this patient group [2, 14].

We investigated whether an in‐hospital delay in patients aged 65 years and older increases the risk of perforation and complications. Since many retrospective studies evaluating the outcome of in‐hospital delay in appendicitis suffer from selection bias, as patients with more symptoms, severe leukocytosis, elevated CRP levels, or suspected complicated appendicitis are operated on more urgently and usually have shorter waiting times, we performed a logistic regression analysis to adjust for risk factors and reduce bias.

## 2 Methods

This study was conducted as a single center retrospective study at the Department of General Surgery, Visceral and Transplant Surgery at the University Hospital Augsburg, Germany. Approval for the study was granted by the Ethics Committee of the Ludwig Maximilians University (LMU), Munich (reference number 23‐0500) and conducted in accordance with the Declaration of Helsinki. The manuscript was prepared in accordance with the STROBE guidelines [22].

### 2.1 Study Population and Definitions

We identified all patients aged 65 years and older who underwent emergency surgery for suspected appendicitis at our institution between January 2008 and June 2023 using electronic health records. Perioperative data were retrieved from the institutional electronic database. Complications, comorbidities, operative data, and patient characteristics were extracted, including age, ASA status, BMI, preoperative symptoms, preoperative CRP and leukocyte levels, radiologic findings, intraoperative findings, operating time, percentage of laparoscopic procedures, histopathology results, complication rate, and length of hospital stay. Perforation on imaging was documented when a perforation was described in the written imaging report. Perforation as an endpoint was defined as histopathologically proven perforation in the appendectomy specimen. Complication rate was defined as the percentage of patients who experienced a perioperative complication. Patients who were initially treated conservatively with antibiotics and/or interventional drainage were not included in the analysis.

### 2.2 Surgical Intervention

Laparoscopic appendectomy was performed using a three‐trocar technique (one 10‐mm trocar for the camera, one 5‐mm trocar, and one 12‐mm trocar). Open appendectomy was performed with an oblique McBurney incision and a muscle‐splitting technique. Depending on the surgeon's preference and intraoperative findings, conversion from laparoscopic to open surgery was performed either via a midline incision or via an oblique incision in the right lower quadrant. In laparoscopic procedures, the appendix was transected using a laparoscopic stapler. Alternatively, in open procedures, either a ligation of the base with transection and inversion of the appendix stump was performed or a stapler was used to transect the base of the appendix.

### 2.3 Statistical Analysis

Continuous data are presented as mean ± standard deviation or median with interquartile range, depending on distribution. Categorical data are presented as numbers with percentages. Continuous variables were compared using the independent *t*‐test or the Mann–Whitney U test depending on distribution. Categorical data were compared using the χ^2^ test. Fisher's exact test was used for categorical data when the requirements for the χ^2^ test were not met. A two‐sided *p* < 0.05 was considered significant. Logistic regression was used to adjust for *American Society of Anesthesiologists* (ASA) score, diabetes, intake of anticoagulants, previous abdominal surgery, body mass index (BMI), white blood cell (WBC) count, C‐reactive protein (CRP) level, presence of guarding on physical examination, duration of symptoms, and suspected perforation on imaging. Odds ratios with a 95% confidence interval (CI) were calculated for each risk factor. An ROC curve was calculated to evaluate the fit of the logistic regression model. Statistical analyses were undertaken using SPSS for macOS, version 29 (IBM, Armonk, New York, USA).

## 3 Results

A total of 525 patients aged 65 years and older underwent appendectomy for suspected appendicitis between January 2008 and June 2023. Patients who were managed conservatively with antibiotics or interventional drainage were excluded from the analysis.

Table 1 shows the clinical and demographic characteristics of the study population. The average age was 74.7 years (range 65–96.5 years). Information on the duration of symptoms was available for 488 of 525 patients. Of these, 199 (40.8%) presented to the emergency department within the first 24 h after the onset of symptoms. 175 (35.9%) of the patients presented more than 48 h after symptom onset and 103 (21.1%) patients presented more than 72 h after symptom onset.

**TABLE 1:** Baseline characteristics.

Characteristic	*n* = 525
Age (mean, SD)	74.7 (6.7)
ASA score	
1	37 (7.0%)
2	257 (49.0%)
3	216 (41.1%)
4/5	15 (2.9%)
Type of surgery	
Laparoscopic	414 (78.9%)
Open	48 (9.1%)
Conversion from laparoscopic to open	63 (12.0%)
Gender	
Female	253 (48.2%)
Male	272 (51.8%)
BMI (mean, SD)	27.1 (± 4.5)
Diabetes	61 (11.6%)
Anticoagulation	220 (41.9%)
Previous abdominal surgery	144 (27.4%)
Abdominal guarding	272 (51.8%)
Duration of symptoms > 48 h	175 (33.3%)
Preoperative WBC count (per nl)	
Mean (SD)	13.7 (5.1)
≤ 10	116 (22.1%)
> 10–≤ 15	233 (44.4%)
> 15–≤ 20	131 (25.0%)
> 20	44 (8.4%)
Unknown	1 (0.2%)
Preoperative CRP level (mg/L)	
Mean (SD)	103 (94.4)
≤ 50	197 (37.5%)
> 50–≤ 100	115 (13.3%)
> 100–≤ 150	70 (13.3.9%)
> 150	141 (26.9%)
Unknown	2 (0.4%)
Suspected perforation on imaging	118 (22.5%)
Diagnosis to surgery (hours)	
mean (SD)	7.36 (± 7.4)
≤ 12 h	4109 (83.9%)
> 12 h	791 (16.1%)
Operating time, minutes (median (IQR))	66 (51.5–84)
Perforation on histopathologic examination	233 (44.4%)
Complication rate	132 (25.1%)
Complications (Clavien–Dindo classification)	
Grade 1 or 2	89 (17.0%)
Grade 3a	12 (2.3%)
Grade 3b	20 (3.8%)
Grade 4a	7 (1.3%)
Grade 4b	1 (0.2%)
Grade 5	3 (0.6%)
Comprehensive complication index (CCI), mean (SD)	6.63 (± 14.0)
Negative appendectomy	30 (5.7%)
Mortality	3 (0.6%)
Length of postoperative stay, days (median (IQR))	4.5 (2.8–7.3)

*Note:* Date are mean ± SD or *n* (%) or median (IQR).

Abbreviations: ASA, American society of anesthesiologists; BMI, body mass index; CRP, C‐reactive protein; WBC, white blood cell.

In terms of diagnostic workup, abdominal ultrasound was performed in 505 (96.2%) patients and computed tomography in 184 (35.0%) patients. A total of 170 (32.4%) patients underwent ultrasound examination and a CT scan.

The mean waiting time from diagnosis to surgery was 407 min (6.8 h). The mean waiting from hospital admission to surgery was 572 min (9.5 h). In 185 (35.2%) patients, the waiting time from diagnosis to surgery was more than 6 h, and in 70 patients (13.3%) the waiting time was more than 12 h.

Laparoscopic surgery was performed in 414 (78.9%) patients. A total of 48 (9.1%) patients underwent open surgery, and 63 (12.0%) patients required conversion from laparoscopic to open surgery.

Histopathologic examination revealed the presence of an appendicitis in 478 of 525 specimens (91.0%). Neoplasms, including both benign adenomas and malignant tumors, were present in 34 of 525 patients (6.5%). Cecal perforation without involvement of the appendix was found in 2 cases. There were no pathological findings in 30 of 525 cases (negative appendectomy rate 5.7%). An appendicolith was diagnosed in 14 cases (2.7%).

### 3.1 Risk Factors for Perforation and Complication

The perforation rate was 44.4% (233 of 525) and the complication rate was 25.1% (132 of 525). Most complications (89 of 312) were classified as minor complications Clavien–Dindo Grade 1 or 2. The average comprehensive complication index CCI was 6.6 and the mortality rate was 0.6% (3 out of 525).

Logistic regression analyses were performed for the endpoints complication and perforation to adjust for potential risk factors. The ROC curves calculated to evaluate the logistic regression model did show a good fit of the model (Figures S1 and S2).

Risk factors for perforation in the multivariable analysis were markedly elevated CRP levels with an OR of 1.72 for CRP levels 50–100 mg/mL (5% CI 1.03–2.86; *p* = 0.038) and with an OR of 4.87 (95% CI 2.65–8.97; *p* < 0.001) for CRP levels > 100 mg/L‐150 mg/L. An in‐hospital delay of more than 12 h compared to an in‐hospital of less than 12 h was not associated with an increased risk for perforation (OR 0.96; 95% CI 0.55–1.70; *p* = 0.90) (Table 2).

**TABLE 2:** Risk factors for perforation in elderly patients—multivariable analysis.

Risk factor	OR	95% CI	*p* (multivariable)
ASA‐score	0.80	0.57–1.1	0.17
Male sex	0.93	0.63–1.37	0.73
BMI ≥ 30 kg/m^2^	0.90	0.58–1.41	0.66
Anticoagulation	1.32	0.86–2.05	0.21
Diabetes	1.00	0.55–1.83	0.96
Previous abdominal surgery	1.11	0.72–1.71	0.63
Symptom onset > 48 h	0.76	0.50–1.18	0.22
Abdominal guarding	0.83	0.57–1.22	0.34
Suspected perforation on imaging	1.50	0.95–2.39	0.09
WBC count (/nl)			
≤ 10	—	—	—
> 10–≤ 15	0.89	0.54–1.47	0.65
> 15–≤ 20	1.24	0.71–2.15	0.45
> 20	1.16	0.52–2.55	0.72
CRP level (mg/L)			
≤ 50	—	—	—
> 50–≤ 100	1.72	1.03–2.86	0.038
> 100–≤ 150	4.87	2.65–8.97	< 0.001
> 150	4.84	2.83–8.27	< 0.001
In‐hospital delay > 12 h	0.96	0.55–1.70	0.90

Abbreviations: ASA, American society of anesthesiologists; BMI, body mass index, CRP, C‐reactive protein; WBC, white blood cell.

The intake of anticoagulants or platelet inhibitors (OR 1.68; 95% 1.03–2.74; *p* = 0.04), a suspected perforation on preoperative imaging (OR 2.06; 95% CI 1.26–3.38 M; *p* = 0.004), and a CRP level of more than 150 mg/L (OR 3.05; 95% CI 1.68–5.54; *p* < 0.001) were associated with an increased risk for complications in the multivariable analysis (Table 3). An in‐hospital delay of more than 12 h was not associated with an increased complication rate compared to an in‐hospital delay of less than 12 h (OR 0.93; 95% CI 0.49–1.76; *p* = 0.83). In addition to the analyses with a 12‐h cutoff for in‐hospital delay, we conducted analyses with hourly consideration of in‐hospital delay. These yielded similar results (Tables S1 and S2).

**TABLE 3:** Risk factors for complication in elderly patients—multivariable analysis.

Risk factor	OR	95% CI	*p* (multivariable)
ASA score	1.42	0.98–2.07	0.07
Male sex	1.11	0.71–1.73	0.66
BMI ≥ 30 kg/m^2^	0.70	0.41–1.19	0.18
Anticoagulation	1.68	1.03–2.74	0.04
Diabetes	1.33	0.69–2.56	0.40
Previous abdominal surgery	1.32	0.82–2.13	0.26
Symptom onset > 48 h	0.89	0.55–1.44	0.63
Abdominal guarding	0.71	0.49–1.09	0.12
Suspected perforation on imaging	2.06	1.26–3.38	0.004
WBC count (/nl)			
≤ 10	—	—	—
> 10–≤ 15	0.95	0.53–1.69	0.85
> 15–≤ 20	0.98	0.51–1.87	0.95
> 20	1.83	0.79–4.23	0.16
CRP level (mg/L)			
≤ 50	—	—	—
> 50–≤ 100	1.13	0.60–2.11	0.71
> 100–≤ 150	1.66	0.83–3.33	0.15
> 150	3.05	1.68–5.54	< 0.001
In‐hospital delay > 12 h	0.93	0.49–1.76	0.83

Abbreviations: ASA, American society of anesthesiologist; BMI, body mass index; CRP, C‐reactive protein; WBC, white blood cell.

## 4 Discussion

Although many studies have investigated the safety of an in‐hospital delay in acute appendicitis, this is one the first studies to address the safety of delayed surgery in the elderly patients. The paucity of data regarding older patients in combination with the often higher rates of complications and perforations in this patient population led to a more cautious stance or lack of guideline recommendations on delayed surgery in this patient population.

A delay of 12 h or more compared to surgical treatment within 12 h was neither associated with an increased risk of perforation nor complications in this study.

These findings in elderly patients are consistent with the results from retrospective and prospective studies that have investigated the safety of in‐hospital delay for acute appendicitis in adult patients [9, 12, 13]. Djik et al. analyzed 45 observational cohort studies including patients of all ages in a meta‐analysis and found no higher risk for complicated appendicitis when appendectomy was delayed by 7–12 h or 13–24 h [8]. Our results in combination with previous studies, investigating all age groups, suggest that older patients with acute appendicitis should not be treated differently with regard to delayed surgery.

Unfortunately, many retrospective studies that have evaluated the safety of delayed surgery—including most of the studies analyzed in the meta‐analysis mentioned above—suffer from selection bias because clinical, laboratory, and radiological signs of complicated disease, such as guarding, marked leukocytosis, elevated CRP levels, or suspected complicated disease on imaging often prompt more urgent surgical intervention. Therefore, we reduced selection bias adjusting for variables that would lead to early surgery in the logistic regression analysis. In addition, we included the duration of symptoms in the multivariable analysis, a possible risk factor for perforation, which is often not considered studies [23].

The perforation rate in our study population was 44.4%, which is in line with the results from subgroups of older patients in other studies (35%–70%) but differs greatly from studies reporting perforation rates across all age groups [2, 4, 5, 9, 19, 20]. A preoperative predictor of appendiceal perforation was a CRP level > 100 mg/L.

The complication rate among elderly patients in this study was 25.1% (132 of 525). Most patients only experienced minor complications and the mortality in our study population was 0.6%. We found that anticoagulant use, suspected perforation on imaging, and elevated CRP levels were independent risk factors for complications in elderly patients. Apart from the higher mean age, perforation, and complication rates, the patient population in our study differs from typical populations of studies investigating treatment options and delayed treatment in acute appendicitis. Of clinical importance is the higher rate of previous abdominal surgery in older patients, which can make laparoscopic appendectomy more difficult and may explain the relatively high rate of open appendectomy and conversion to open surgery.

In this study, the rate of appendiceal neoplasia including benign and malignant lesions was 6.5%. Renteria et al. reported a rate of 3% incidental malignant tumors of the appendix in patients over 60 years of age compared to only 1.5% in patients under 60 years of age, and 11% (7 of 64) of patients who underwent interval appendectomy after initial nonoperative treatment and had a neoplasm in the appendix [24].

Naar et al. reported an incidence of 1.5% for malignant appendiceal lesions and identified the combination of age over 40 years and an appendix diameter greater than 10 mm as a risk factor for the presence of an appendiceal malignancy with on OR of 3.03.

The finding that a delay of up to 12 h does not result in an increased risk of perforation or complications may be of particular value for patients with preoperative imaging suggestive of a neoplasm as it opens a window of opportunity for more comprehensive work‐up and surgical planning if necessary. For patients taking anticoagulants, delaying surgery allows for discontinuation of medication or correction of abnormal coagulation values.

Despite the seemingly high rate of perforation in older patients, reported in many studies, Andersson et al. showed that the incidence of perforated appendicitis is not higher in elderly patients than in young patients, when looking at the overall population [25]. The authors even took into account that older patients represent a potentially smaller “at‐risk population” for appendicitis and adjusted for the age‐related increase in the proportion of individuals who have already undergone appendectomy during their lifetime and found that only the relative incidence of perforated appendicitis appears to be higher in elderly patients which is caused by a reduction in the incidence of nonperforated appendicitis and the overall incidence of appendicitis in elderly patients is significantly lower compared to younger patients [25]. The finding supports the assumption that perforating and nonperforating appendicitis might be different entities. Based on epidemiologic data and time series analysis as well as immunologic studies, it has been postulated that perforating and nonperforating appendicitis have a different underlying pathophysiology [5, 8, 10, 11].

Although multivariable analysis may reduce selection bias, given the retrospective nature of this study, documentation bias, to which most retrospectives are susceptible, cannot be excluded. Although radiological signs of perforation have been defined in the literature, the lack of a standardized definition for suspected perforation on imaging could be considered a weakness of this study. The reporting of perforation in the written ultrasound or CT report was solely at the discretion of the examining radiologist or physician based on their findings [26]. To enable better comparability of the results, a 12‐h cutoff was applied for the analysis, which is used in several publications and guidelines [8, 9, 14]. However, as there is little evidence to support the use of a 12‐h cutoff, an hourly analysis was also performed to determine the influence of waiting time on perforation and complication rates.

A particular strength of this study compared to the published prospective trials is the analysis of real‐world data. Not only did we include patients with high inflammatory values levels and with suspected perforation on preoperative imaging, but the collected data also represent a time span of 15 years. Furthermore, the preoperative examinations were performed by different radiologists, physicians, and surgeons with a range of skills and experience.

This study provides evidence for the safety of in‐hospital delay of surgery in acute appendicitis in patients aged 65 years and older. Despite this result, it should always be kept in mind that early surgical treatment shortens the time of discomfort for patients and unnecessary delay in treatment should be avoided whenever possible.

## 5 Conclusion

In this large retrospective study, an in‐hospital delay of surgery of 12 h was not associated with an increased rate of perforation rate or complications in patients aged 65 and older after adjustment for risk factors. Suspected perforation on diagnostic imaging, anticoagulant use, and markedly elevated preoperative CRP levels were associated with an increased risk of complications and should be taken into account when considering the delay of surgery.

## Author Contributions

**Matthias C. Schrempf:** conceptualization, formal analysis, writing – original draft, investigation, methodology, project administration. **Stefan Schiele:** formal analysis, methodology, software. **Matthias Anthuber:** supervision, writing – review and editing. **Lena Anthuber:** conceptualization, writing – review and editing. **Michael Hoffmann:** writing – review and editing. **Florian Sommer:** writing – review and editing, resources. **Andrea Mair:** conceptualization, data curation, formal analysis, writing – original draft, investigation.

## Funding

This is an academic investigator‐initiated study without external funding.

## Conflicts of Interest

The authors declare no conflicts of interest.

## Supporting information


**Figure S1:** ROC curve predicted probability for perforation.

**Figure S2:** ROC curve predicted probability for complication.

**Table S1:** Risk factors for perforation in elderly patients—multivariable analysis—in‐hospital delay analyzed per hour. ASA, American society of anesthesiologists; BMI, body mass index; CRP, C‐reactive protein; WBC, white blood cell.

**Table S2:** Risk factors for complication in elderly patients—multivariable analysis—in‐hospital delay analyzed per hour. ASA, American society of anesthesiologists; BMI, body mass index; CRP, C‐reactive protein; WBC, white blood cell.
