# Supplementary material for: Safety of In‐Hospital Delay of Appendectomy in Elderly Patients—A Retrospective Analysis of 525 Consecutive Patients Aged 65 and Older Undergoing Surgery for Suspected Appendicitis
Source: World J Surg. 2025 Dec 5;50(1):130–6. doi: 10.1002/wjs.70178 (PMC12831526; doi:10.1002/wjs.70178)
Supplement: Supplementary file 1 — Figure S1: ROC curve predicted probability for perforation. [file WJS-50-130-s003.docx]

Supplemental Fig. 1: ROC curve predicted probability for perforation


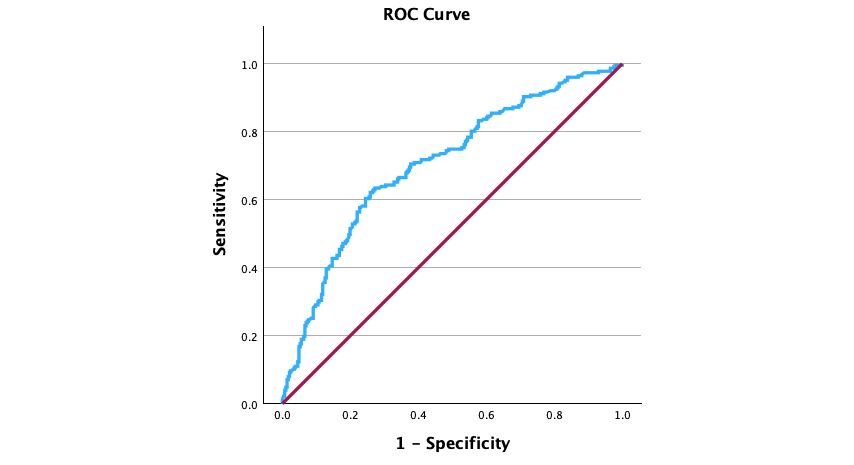


**Area Under the Curve**

Test Result Variable(s): Predicted probability for perforation

| Area | Std. Error^a^ | Asymptomatic Sig.^b^ | Asymptotic 95% Confidence Interval | |
| --- | --- | --- | --- | --- |
|  |  |  | Lower Bound | Upper Bound |
| .705 | .023 | <0.0001 | .660 | .750 |

1. Under the nonparametric assumption
2. Null hypothesis: true area = 0.5
