# Supplementary material for: Safety of In‐Hospital Delay of Appendectomy in Elderly Patients—A Retrospective Analysis of 525 Consecutive Patients Aged 65 and Older Undergoing Surgery for Suspected Appendicitis
Source: World J Surg. 2025 Dec 5;50(1):130–6. doi: 10.1002/wjs.70178 (PMC12831526; doi:10.1002/wjs.70178)
Supplement: Supplementary file 3 — Table S1: Risk factors for perforation in elderly patients—multivariable analysis—in‐hospital delay analyzed per hour. ASA, American society of anesthesiologists; BMI, body mass index; CRP, C‐reactive protein; WBC, white blood cell. [file WJS-50-130-s004.docx]

| Risk factor | OR | 95% CI | p (multivariable) |
| --- | --- | --- | --- |
| ASA-Score | 0.79 | 0.57 – 1.1 | 0.16 |
| Male sex | 0.94 | 0.64 – 1.39 | 0.76 |
| BMI ≥30 | 0.90 | 0.58 – 1.41 | 0.65 |
| Anticoagulation | 1.31 | 0.85 – 2.02 | 0.23 |
| Diabetes | 0.99 | 0.54 – 1.82 | 0.98 |
| Previous abdominal surgery | 1.11 | 0.72 – 1.70 | 0.64 |
| Symptom onset > 48 h | 0.76 | 0.49 – 1.17 | 0.21 |
| Abdominal guarding | 0.84 | 0.57 – 1.22 | 0.35 |
| Suspected perforation on imaging | 1.50 | 0.94 – 2.39 | 0.09 |
| WBC count (/nl)  ≤ 10  > 10 – ≤ 15  > 15 – ≤ 20  > 20 | -  0.90  1.24  1.16 | -  0.55 – 1.48  0.71 – 2.15  0.53 – 2.57 | -  0.67  0.45  0.71 |
| CRP level (mg/l)    ≤ 50  > 50 – ≤ 100  > 100 – ≤ 150  > 150 | -  1.74  4.88  4.88 | -  1.04 – 2.90  2.65 – 8.98  2.86 – 8.34 | -  0.034  <0.001  <0.001 |
| In-hospital delay (per hour waiting time) | 1.01 | 0.98 – 1.04 | 0.66 |
| BMI body mass index, ASA American Society of Anaesthesiologists, WBC white blood cell, CRP C-reactive protein | | | |

Supplementary Table 1 Risk factors for perforation in elderly patients – multivariable analysis - in-hospital delay analyzed per hour
