# Supplementary material for: Safety of In‐Hospital Delay of Appendectomy in Elderly Patients—A Retrospective Analysis of 525 Consecutive Patients Aged 65 and Older Undergoing Surgery for Suspected Appendicitis
Source: World J Surg. 2025 Dec 5;50(1):130–6. doi: 10.1002/wjs.70178 (PMC12831526; doi:10.1002/wjs.70178)
Supplement: Supplementary file 4 — Table S2: Risk factors for complication in elderly patients—multivariable analysis—in‐hospital delay analyzed per hour. ASA, American society of anesthesiologists; BMI, body mass index; CRP, C‐reactive protein; WBC, white blood cell. [file WJS-50-130-s001.docx]

| Risk factor | OR | 95% CI | p (multivariable) |
| --- | --- | --- | --- |
| ASA-Score | 1.43 | 0.98 – 2.08 | 0.063 |
| Male sex | 1.10 | 0.70 – 1.72 | 0.68 |
| BMI ≥ 30kg/m^2^ | 0.70 | 0.41 – 1.19 | 0.18 |
| Anticoagulation | 1.69 | 1.04 – 2.76 | 0.035 |
| Diabetes | 1.34 | 0.70 – 2.59 | 0.38 |
| Previous abdominal surgery | 1.32 | 0.82 – 2.14 | 0.25 |
| Symptom onset > 48 h | 0.64 | 0.55 – 1.45 | 0.64 |
| Abdominal guarding | 0.71 | 0.46 – 1.09 | 0.12 |
| Suspected perforation on imaging | 2.08 | 1.27 – 3.41 | 0.004 |
| WBC count (/nl)  ≤ 10  > 10 – ≤ 15  > 15 – ≤ 20  > 20 | -  0.94  0.98  1.82 | -  0.53 – 1.67  0.51 – 1.87  0.78 – 4.20 | -  0.83  0.95  0.16 |
| CRP level (mg/l)    ≤ 50  > 50 – ≤ 100  > 100 – ≤ 150  > 150 | -  1.12  1.66  3.05 | -  0.60 – 2.10  0.83 – 3.34  1.68 – 5.53 | -  0.73  0.15  <0.001 |
| In-hospital delay > 12 h (per hour waiting time) | 0.99 | 0.96 – 1.02 | 0.57 |
| BMI body mass index, ASA American Society of Anaesthesiologists, WBC white blood cell, CRP C-reactive protein | | | |

Supplementary Table 2 Risk factors for complication in elderly patients – multivariable analysis - in-hospital delay analyzed per hour
